# ASO Author Reflections: Trends of Axillary Treatment in Sentinel Node-Positive Breast Cancer Patients Undergoing Mastectomy

**DOI:** 10.1245/s10434-023-13785-w

**Published:** 2023-06-28

**Authors:** Eline Verreck, Julia van Steenhoven, Thijs van Dalen

**Affiliations:** 1 Utrecht University, Utrecht, The Netherlands; 2 Surgery, Diakonessenhuis Utrecht, Utrecht, The Netherlands; 3 Pathology, Universitair Medisch Centrum Utrecht, Utrecht, The Netherlands; 4 Surgery, Erasmus MC, Rotterdam, The Netherlands; 5 Surgery, University Medical Centre Utrecht, Utrecht, The Netherlands

## Past

During the last decade, the necessity to perform axillary lymph node dissection (ALND) in clinically node-negative (cN0) patients with sentinel lymph node metastases (SLN+) is debated worldwide. For patients with early breast cancer undergoing breast-conserving therapy (BCT), the ACOSOG-Z0011 trial has allowed for avoidance of unnecessary axillary lymph node dissection (ALND) in patients with 1–2 positive SLNs without compromising outcome.^1^ The AMAROS trial investigated axillary radiotherapy (RT) versus ALND in patients with tumor-affected SLNs and showed that axillary RT could serve as a safe alternative to ALND with less morbidity.^2^ Since 2012, both national and international guidelines suggest no further axillary treatment in patients meeting the Z0011 criteria or advocate regional radiotherapy (RT) as alternative therapy for ALND. As patients treated with mastectomy were not included in the Z0011 and underrepresented in the AMAROS trial (with only 18% receiving mastectomy), evidence regarding the omission of ALND in this subset of patients is scarce. In the present study, we aimed to explore patterns of axillary treatment in Dutch patients with SLN+ breast cancer undergoing mastectomy.

## Present

In our study, which included 10,633 SLN+ patients with cT1–3 breast cancer who underwent mastectomy, the frequency of ALND decreased from 78% in 2009 to 10% in 2018. In patients with micrometastasis (N1mi) and isolated tumor cells (N0itc), ALND was soon abandoned, whereas 20% of patients diagnosed with ≥ N1a disease still underwent ALND at the end of the study period. Over the same period, postmastectomy radiotherapy (PMRT) increased from 4 to 49%. The increase of PMRT was merely seen in patients with ≥ N1a disease, from 2 to 70%, showing ALND is mostly replaced with PMRT in this group of patients. Besides the association between the degree of metastatic lymph node involvement and the performance of ALND, factors such as age, differentiation grade, tumor subtype, and hospital type affected the performance of ALND. Factors associated with a higher chance of ALND performance were age < 40 years, lobular tumor type, basal-like tumor type, receiving chemotherapy, and treatment outside an academic institution.

## Future

The present data illustrate a strong reduction in the performance of ALND in SLN+ patients undergoing mastectomy. Recently, the results of the SINODAR-ONE trial, which included a substantial proportion of SLN+ patients treated by mastectomy (25%) showed excellent regional control among SLN+ patients who received no further local treatment.^3^ Currently, there are two more ongoing trials evaluating the oncological safety of omitting axillary therapy in node-positive patients treated by mastectomy (the POSNOC trial and the SENOMAC trial).^4,5^ The observed tendency among clinicians to deescalate axillary surgery in the present study suggests that clinicians felt comfortable enough to avoid ALND in this subset of patients despite the limited evidence. Long-term follow-up results from the aforementioned trials, possibly supported by outcome data from national registries, may not only provide more robust support for avoiding surgical staging of axillary lymph nodes, but may also help to better delineate the indication for radiotherapy when ALND is omitted.
